# Hard X-rays as *pump* and *probe* of atomic motion in oxide glasses

**DOI:** 10.1038/s41598-017-04271-x

**Published:** 2017-06-21

**Authors:** B. Ruta, F. Zontone, Y. Chushkin, G. Baldi, G. Pintori, G. Monaco, B. Rufflé, W. Kob

**Affiliations:** 1 ESRF- The European Synchrotron, F-38043 Grenoble, France; 2 Institut Lumière Matière, UMR5306 Université Lyon 1-CNRS, Université de Lyon, 69622 Villeurbanne Cedex, France; 3 Dipartimento di Fisica, Trento University, I-38123 Povo, Trento Italy; 4 Laboratoire Charles Coulomb (L2C), UMR 5221 CNRS-Université de Montpellier, Montpellier, FR-34095 France

## Abstract

Nowadays powerful X-ray sources like synchrotrons and free-electron lasers are considered as ultimate tools for probing microscopic properties in materials. However, the correct interpretation of such experiments requires a good understanding on how the beam affects the properties of the sample, knowledge that is currently lacking for intense X-rays. Here we use X-ray photon correlation spectroscopy to probe static and dynamic properties of oxide and metallic glasses. We find that although the structure does not depend on the flux, strong fluxes do induce a non-trivial microscopic motion in oxide glasses, whereas no such dependence is found for metallic glasses. These results show that high fluxes can alter dynamical properties in hard materials, an effect that needs to be considered in the analysis of X-ray data but which also gives novel possibilities to study materials properties since the beam can not only be used to *probe* the dynamics but also to *pump* it.

## Introduction

Usually the interaction between X-rays and matter is weak and therefore they are an excellent probe to study the properties of materials^1^. However, the tremendous increase in brilliance of modern X-ray sources such as third-generation synchrotrons and free-electron lasers allows now to use photon fluxes that are so high that one cannot assume any more that the probe beam does not affect the properties of the sample. It is therefore important to obtain a solid understanding on how this strong flux influences the measurements since only this knowledge will allow a correct interpretation of the obtained results.

As it has been documented before, X-rays can in fact induce chemical rearrangements in soft materials like polymers and biological samples^2^. Here the ionizing action of the radiation can modify the structure of the system and even result in its complete disruption. In these cases two main processes have been identified^3, 4^. A first primary damage occurs on femtosecond timescales, and is related to the creation of free radicals by photoelectric absorption and Compton scattering^3^. The diffusion of these radicals can then lead to a secondary damage through the formation of additional radicals and the breaking of chemical bonds. As this second process occurs on a timescale of microseconds to milliseconds at room temperature, it can be largely suppressed by working at cryogenic temperatures^5^. Although it is impossible to completely remove the radiation damage, studies on protein crystallography show that it is nevertheless possible to get unique intrinsic properties of a given system^6–8^. For the case of soft materials the radiation damage can limit the achievable resolution^9^. However, this problem can be considerably alleviated by means of fast single shot exposure at high brilliance and fast X-ray sources as free-electron lasers^10, 11^.

In hard condensed matter X-rays can affect the system through three main mechanisms: Radiolysis, knock-on events and electron rearrangements^12, 13^. The first process occurs under UV and X-rays irradiation and leads to the formation of electron-hole pairs and atomic rearrangements. The second effect manifests itself via the displacements of atoms by collision processes that lead to the formation of vacancy-interstitial pairs, such as Frenkel defects^13^. This process usually requires energies as high as 100 keV in order to break atomic bonds in the irradiated materials. The third process are electron rearrangements which occur in presence of pre-existing (natural or induced) defects that serve as electron and hole trap.

Recently Leitner *et al*. investigated for the first time the interaction between hard X-rays and crystalline materials by looking directly at its consequence on the atomic diffusion^14^. That study was done by using X-ray Photon Correlation Spectroscopy (XPCS)^15^, a technique which has emerged as a powerful tool to probe the atomic motion in hard crystalline^16^ and amorphous materials^17^. By collecting series of diffuse scattering patterns with coherent X-rays, XPCS measures the atomic motion through the temporal intensity fluctuations of the speckles generated by the interference of the waves scattered by the atoms in the material. The detailed analysis of Leitner and co-workers shows that the radiation impinging on crystalline alloys can be treated in the linear response regime, i.e. the perturbation due to the beam is negligible^14^.

Here we show that things change completely for the case of simple oxide glasses such as vitreous silica and germania, where the X-rays generate a non-trivial stationary dynamics. In strong contrast to this behaviour, we find that the intrinsic dynamics of metallic glasses is not affected by the X-rays as in crystalline alloys^14^. Differences in electronic properties and the way electronic excitations couple to phonons are probably the reason for the different behaviour of these two classes of amorphous materials.

This unexpected interaction of the X-rays with hard materials has so far been totally neglected in the interpretation of X-ray data and resembles the one recently observed with transmission electron microscopy^18^. This suggests that X-rays can be also used as a novel type of *pump and probe* experiment, allowing to get physical properties of materials which cannot be achieved by means of any other technique.

## Results

The effect of hard X-rays on the atomic motion of a given material can be studied by comparing dynamical measurements taken for different sample positions and incoming intensities selected by inserting X-ray attenuators along the beam path. Each attenuator is made of a polished silicon single crystal and leads to a decrease of the X-ray flux, *F*, by a factor ≈1/e at 8.1 keV. We have measured the dynamics with no attenuators (*F*_0_ ≈ 1 · 10^11^ ph/s) and with filters of different thickness: ≈80 μm thick (*F*_1_≈3 · 10^10^ ph/s), ≈160 μm thick (*F*_2_ ≈ 1.2 · 10^10^ ph/s), and ≈240 μm thick (*F*_3_ ≈ 3.6 · 10^9^ ph/s).

**Figure 1:**
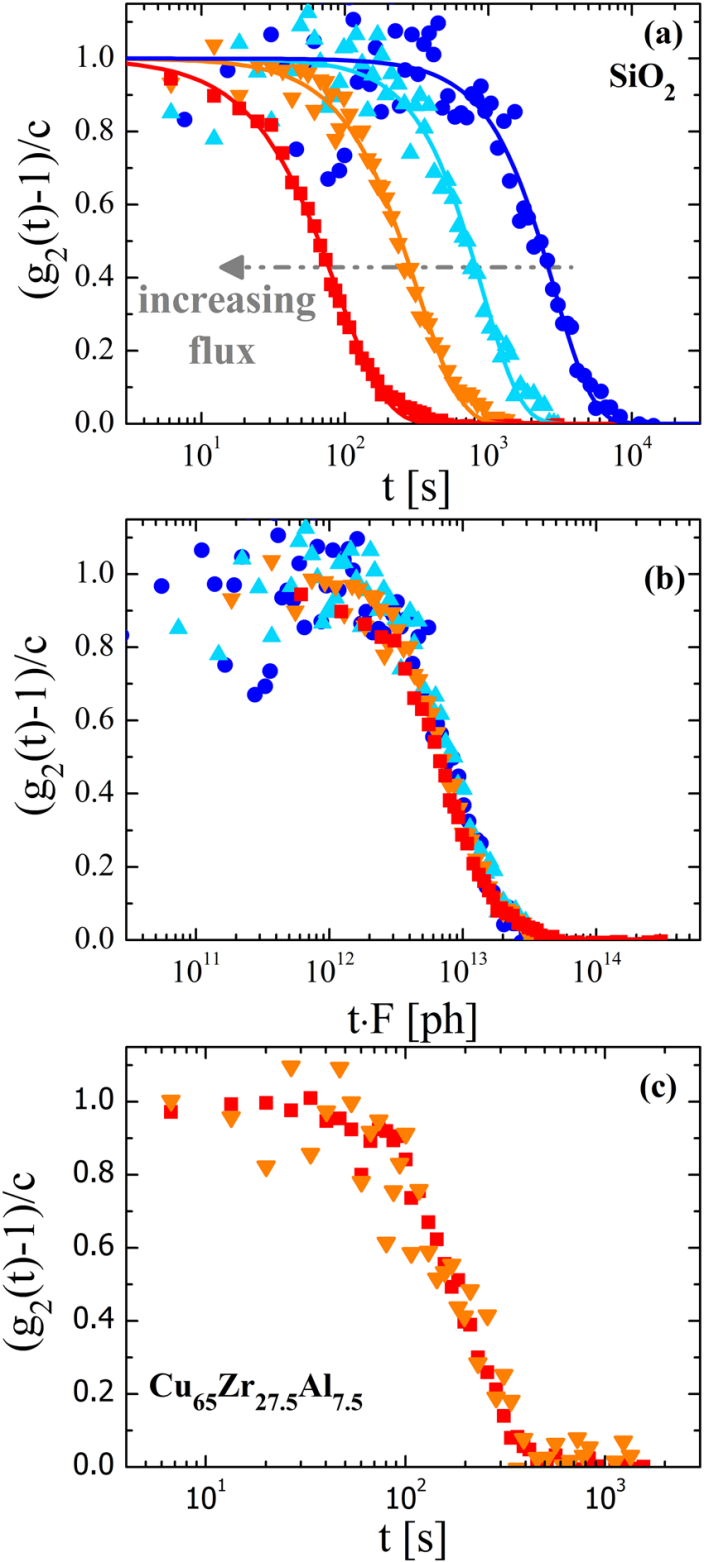
Relaxation dynamics of the atoms as a function of the incident X-ray beam intensity. (**a**) Normalized intensity auto-correlation functions measured in vitreous silica at T = 295 K and wave-vector Q_p_ = 1.5 Å^−1^ for different intensities of the flux of the X-ray beam. From left to right: F_0_ ≈ 1 · 10^11^ ph/s (red squares), F_1_ ≈ 3 · 10^10^ ph/s (orange down-triangles), F_2_ ≈ 1.2 · 10^10^ ph/s (cyan up-triangles) and F_3_ ≈ 3.6 · 10^9^ ph/s (blue circles). Lines are fits with a Kohlrausch-Williams-Watts function. The corresponding decay times are reported in Fig. 2d while the shape parameter β is found to be ~1.4 ± 0.1, independent on the incoming flux. (**b**) Same data rescaled by the incoming flux. (**c**) Normalized intensity auto-correlation functions measured in Cu_65_Zr_27.5_Al_7.5_ metallic glass at T = 413 K and Q_p_ = 2.5 Å^−1^ for F_0_ ≈ 1 · 10^11^ ph/s (red squares), and F_1_ ≈ 3 · 10^1^ ph/s (orange down-triangles).

Figure 1a shows the normalized intensity autocorrelation function g_2_(Q,t) of SiO_2_ at T = 295 K and for a wave-vector Q_p_ = 1.5 Å^−1^ corresponding to the position of the maximum in the structure factor. The function g_2_(Q, t) is directly related to the density fluctuations in the material and thus provides information on its relaxation dynamics^15^. Lines in the figure are fits using the Kohlrausch-Williams-Watts (KWW) function g_2_(Q, t) = 1 + c · exp(−2(t/τ)^β^), where *c* contains information on the experimental contrast and the nonergodicity factor of the glass, *β* describes the shape of the curve, and *τ* is the characteristic decay time^15^.

Surprisingly the correlation functions of vitreous silica display a full decorrelation to zero, even at ambient temperature, thus a temperature that is only 20% of the glass transition temperature T_g_, in agreement with previous results for silicate glasses^19, 20^. However, in marked contrast with these previous studies, the decorrelation on the atomic scale observed here cannot be ascribed to *spontaneous* density fluctuations as it depends strongly on the incident flux of the sample in that the decay shifts toward longer times upon decreasing *F*. Since the decay time increases by about two orders of magnitude when the flux is decreased by the same amount we expect that *τ* is inversely proportional to *F*. This is confirmed by the presence of a master curve in Fig. 1b where we show the same data as in panel (a), but now as a function of the time normalized to the flux. Similar results have been obtained also for GeO_2_ (see Fig. S1), while this effect is absent in metallic glasses (Fig. 1c), in agreement with the work of Leitner and coworkers^14^. As discussed below, the marked difference in the atomic motion of oxide and metallic glasses can be attributed to the different manner how metals and insulators react on the atomic scale to the deposit of the energy carried by the X-rays and the concomitant *real* occurrence of microscopic structural rearrangements in metallic glasses, as confirmed also by several X-ray diffraction studies^21–23^.

**Figure 2:**
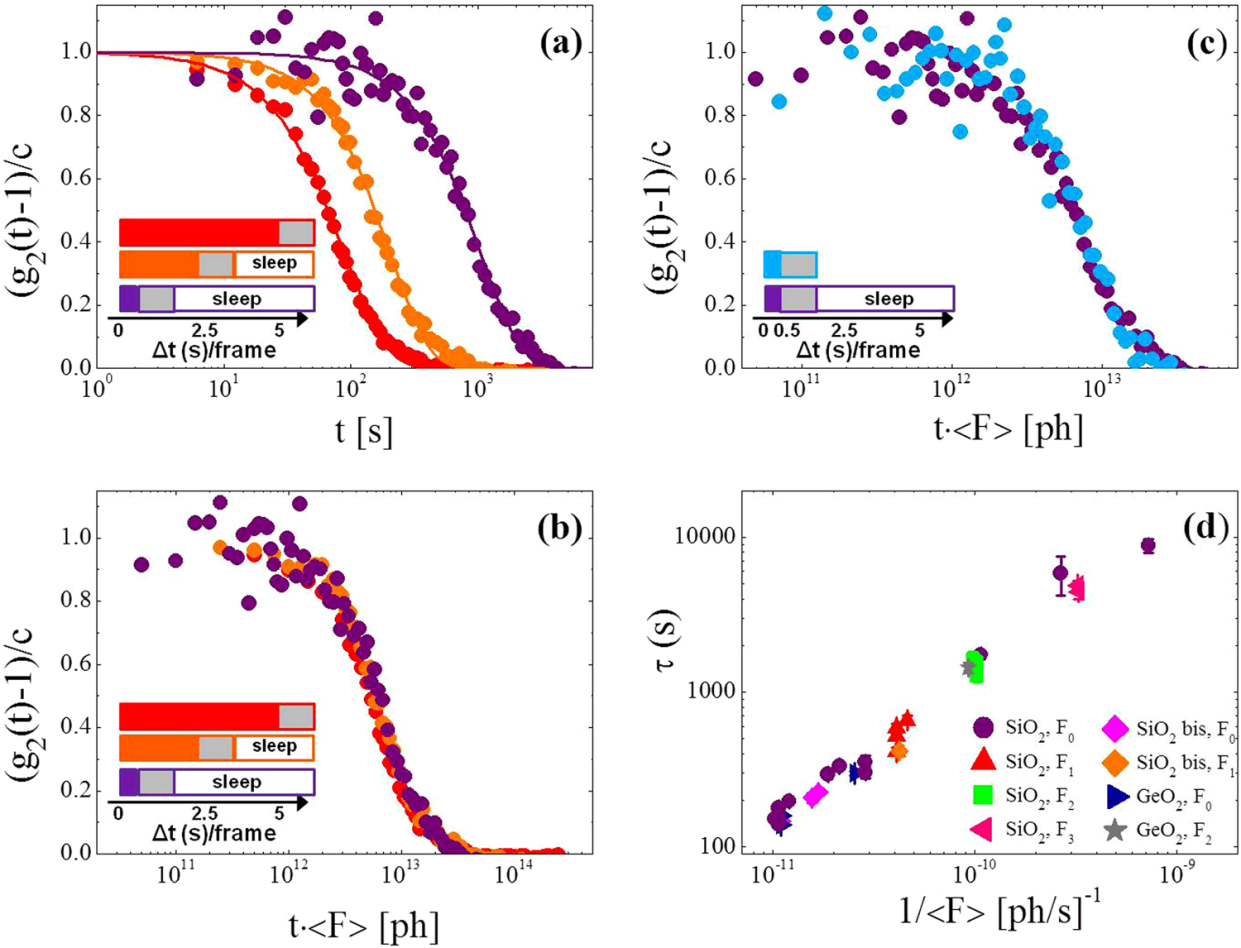
Tuning of the atomic motion. (**a**) Normalized intensity auto-correlation functions measured in vitreous silica at T = 295 K and Q_p_ = 1.5 Å^−1^ with fixed lagtime per frame, Δt = 6.15 s, but with different sleeping times Δt_s_ and exposure times Δt_e_. From left to right: Δt_e_ = 5 s and Δt_s_ = 0 s (red), Δt_e_ = 2.5 s and Δt_s_ = 2.5 s (orange), Δt_e_ = 0.5 s and Δt_s_ = 4.5 s (purple). The legend illustrates the acquisition mode per frame with full coloured boxes for the exposure times Δt_e_ (beam on), empty boxes both for the sleeping times Δt_s_ (beam off) and the constant readout time of the CCD Δt_r_ (beam off, grey boxes). (**b**) Same data as in panel (a) but now normalized by the mean flux <F> = F_0·_Δt_e_/Δt. (**c**) (g_2_(t) − 1)/c measured with Δt_e_ = 0.5 s and Δt_s_ = 4.5 s (same purple data as in panel (a) reported as a function of the time t times the mean flux. The data are compared with the g_2_(t) measured with the same exposure time Δt_e_ = 0.5 s and no sleeping time (Δt_s_ = 0 s, cyan). (**d**) Decay time for different combinations of the incident flux and the lagtime. The data are shown as a function of the inverse mean flux. Also included is data for a second SiO_2_ sample (SiO_2_ bis) and for vitreous GeO_2_. The legend indicates the flux used for each set of measurements.

The observed dependence of the relaxation dynamics on the incident flux suggests that in the absence of such a flux silica glass is in an arrested state, with relaxation times likely too large to be observed during the experimental time scale considered here. We have tested this hypothesis with a set of measurements that had a constant lagtime, *Δt* = 6.15 s, between two consecutive images. This time is the sum of the exposure time Δ*t*_*e*_ (beam on), the sleeping time *Δt*_*s*_ (beam off), and the readout time *Δt*_*r*_ = 1.15 s (beam off), i.e. *Δt* = *Δt*_*e*_ + *Δt*_*s*_ + *Δt*_*r*_. Figure 2a clearly shows that the measured decay time *τ*, increases as we reduce the exposure time. This process follows approximately the relationship: $\tau ={\tau }_{0}\frac{{\rm{\Delta }}t}{\Delta {t}_{e}}$, where *τ*_0_ is the decay time for continuous exposure and negligible readout time (*Δt*_*s*_ = *Δt*_*r*_ = 0). For example, g_2_(Q, t) measured with a continuous exposure of *Δt*_*e*_ = 5 s per frame (red symbols) is approximately one order of magnitude faster than that obtained by using *Δt*_*e*_ = 0.5 s and a sleeping time *Δt*_*s*_ = 4.5 s between frames (purple symbols). This result gives thus evidence that during the sleeping time the sample does not relax on the atomic scale, at least on the scale of 2-3 hours and at the room temperature considered here. As a consequence the measured decay time can thus be expected to be completely controlled by the mean photon flux impinging on the sample between two frames. To test this we plot in Fig. 2b the g_2_(Q, t) of Fig. 2a as a function of the mean flux $< F > ={F}_{0}\frac{{\rm{\Delta }}{t}_{e}}{{\rm{\Delta }}t}$. The almost perfect overlap of the three curves clearly demonstrates that the measured dynamics is indeed triggered by the X-ray beam acting both as a pump and a probe. Another way to see this is to keep the exposure time fixed and to vary the sleeping time (Fig. 2c). We recognize that the duration of the sleeping time does not affect the relaxation time of the system, thus showing that the latter depends only on the mean flux onto the sample (see Fig. S2 for the same scaling for GeO_2_).

The results we just discussed imply that the relaxation time τ of the system is inversely proportional to the mean flux <F>, and thus can be changed basically at will. This is shown in Fig. 2d where we plot τ for a large set of data taken with different parameters (exposure times, sleeping times, and incident intensities) as a function of the mean flux received by the sample in each frame. Also included is data taken in a second SiO_2_ sample and in GeO_2_. It is clear that τ depends linearly on the inverse of the X-ray flux impinging the sample, suggesting a very slow dynamics in absence of X-rays, at least in these two simple oxide glasses. This result is in agreement with the work of Welch *et al*. who reported for similar systems a relaxation time of about ~27 d at ambient temperature^24^.

**Figure 3:**
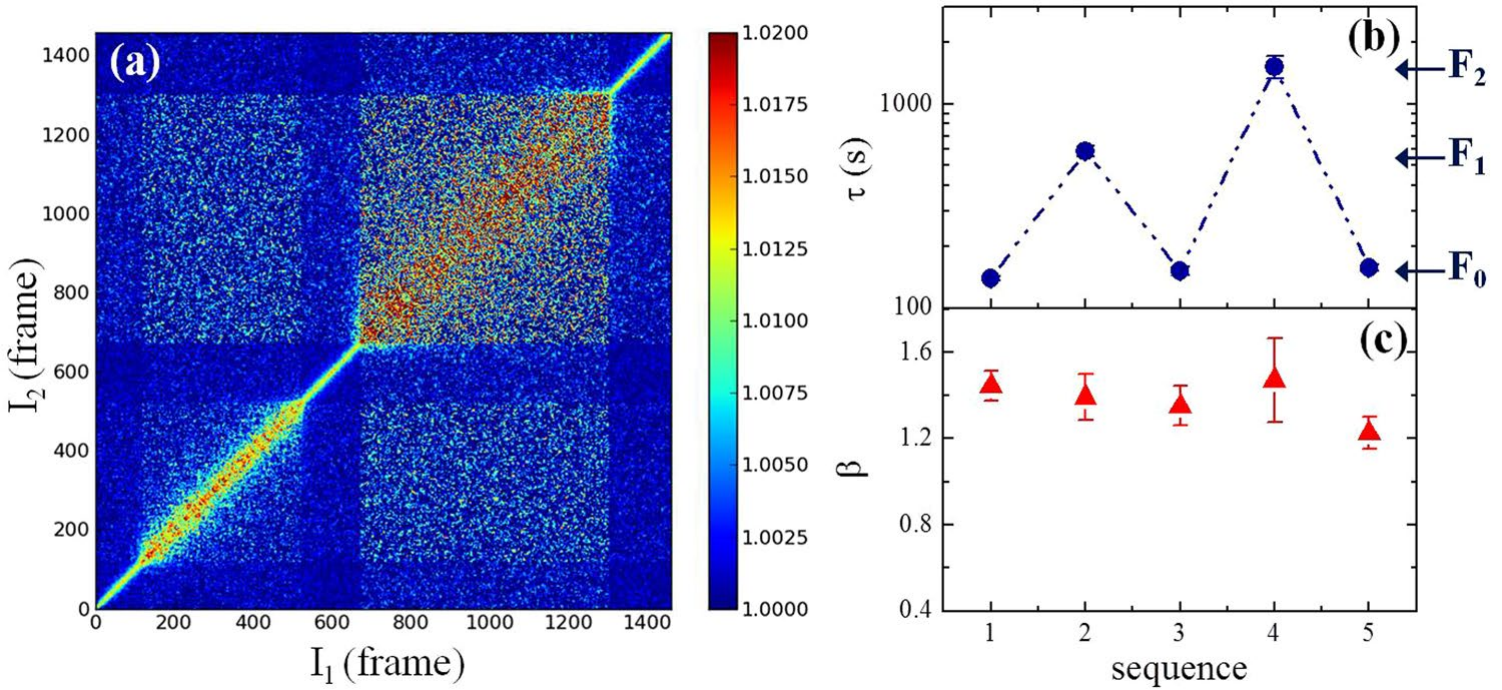
Instantaneous, reversible and stationary dynamics. (**a**) Two-time correlation function measured in vitreous silica at T = 295 K and Q_p_ = 1.5 Å^−1^ by varying the intensity of the incoming flux. Left to right in frame number: F_0_ ≈ 1 · 10^11^ ph/s, F_1_ ≈ 3 · 10^10^ ph/s, F_0_ ≈ 1 · 10^11^ ph/s, F_2_ ≈ 1.2 · 10^10^ ph/s, and F_0_ ≈ 1 · 10^11^ ph/s. Each frame corresponds to Δt = 6.15 s. (**b**) Characteristic decay times τ as a function of the flux intensities used in panel (a). (**c**) Shape parameters β as a function of the flux intensities used in panel (a).

The effect discussed here cannot be classified as standard radiation damage since the induced dynamics is independent on the accumulated dose deposited on a particular sample position (see S.I.). Furthermore, we have found that the decay time can be reversibly modified by simply changing the intensity of the beam. This is demonstrated in Fig. 3a that shows the two-time correlation function (TTCF) measured in SiO_2_ while changing the beam attenuation without stopping the measurement. As explained in ref. 25, each point of the TTCF corresponds to the product of two images acquired at two different times. The effect of the different attenuators is signalled by the abrupt changes in the TTCF profile along the main diagonal whose width is proportional to the decay time of g_2_(Q, t). The higher the incoming intensity, the faster is the dynamics and thus the thinner is the intensity broadening along the diagonal. Interestingly, the decay time changes quickly (in less than one frame, thus ≈6 s) as we insert the corresponding attenuator during data acquisition. In addition, for a fixed incoming intensity the profile of the TTCF remains constant with time thus implying that the corresponding dynamics is stationary and does not depend on the total accumulated dose. By averaging the set of images corresponding to each attenuator we obtain five g_2_(Q,t) whose decay times and shape parameters are reported in Fig. 3b and c. While τ jumps in a reversible way between the values associated with each flux, β remains constant with β = 1.38 ± 0.09, which strongly differs from the stretched exponential decay (i.e. with β < 1) observed in silicates^19, 20^. Similar compressed decays (i.e. with β > 1) have been reported for soft materials and metallic glasses and they could be associated to a strain field in the material generated by a random distribution of slowly-evolving sources of internal stresses^26, 27^.

In view of the presented results one might wonder whether the same X-ray induced dynamics occurs also in previously measured silicates^19, 20^. Unfortunately, insufficient statistics does not allow to perform the same test as in SiO_2_. Even if present, the observed effect can be expected to disappear at high temperatures as the relaxation times measured in the glass transition region of sodium tetrasilicate glass (NS4) gently decrease with increasing temperatures and are found to match macroscopic measurements in the supercooled liquid^19^. It is important to note that despite the flux dependence of the relaxation time, the measured dynamics still *provides* physical information on the probed material. The shape of the correlation functions is compressed in the two simple oxide glasses and in the metallic glass investigated here while stretched in silicates, hence independently of whether the dynamics is induced by the X-ray beam or not.

**Figure 4:**
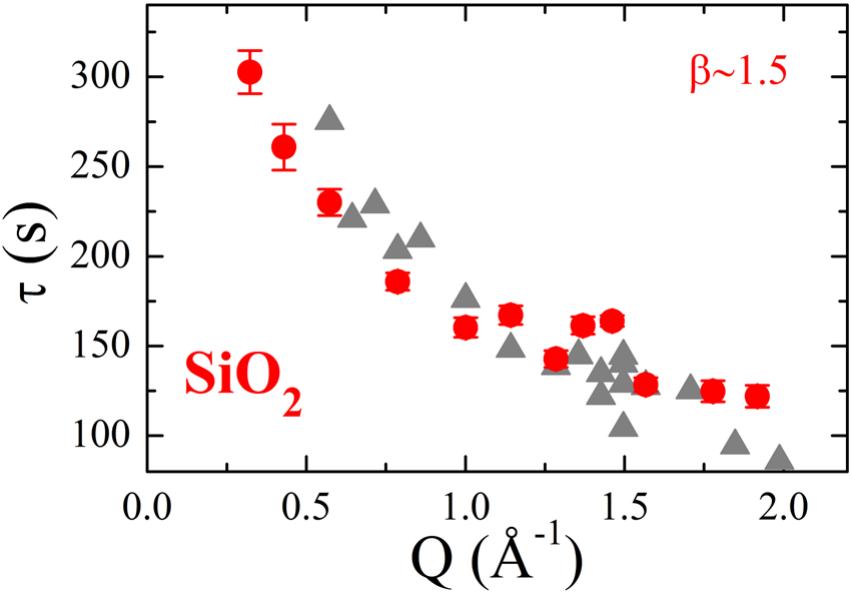
Wave-vector dependence of the X-ray induced dynamics. Wave-vector dependence of the characteristic decay time in vitreous silica measured at T = 295 K and for F_0_ ≈ 1 · 10^11^ ph/s (red circles). The grey triangles are taken with F_1_ ≈ 3 · 10^10^ ph/s and rescaled by the factor 2.74 corresponding to the X-ray intensity difference between the two measurements.

The distinct nature of the probed atomic motion in silica and silicates leads also to a different dependence of the decay time on the wave-vector Q. For SiO_2_ and GeO_2_, τ continuously decreases on increasing Q (Fig. 4 for SiO_2_ and Fig. S3 for GeO_2_), whereas it displays an oscillatory behaviour in NS4^19^ and in the lead silicates studied by Ross and coworkers^20^. It is important to stress that the *incident flux* impinging on the samples is *the same for* both glasses (SiO_2_ and NS4) and *all wave-vectors*. Therefore, the intensity cannot be responsible for the observed differences that should instead be ascribed to the details of the local atomic surroundings. Indeed, at low Qs τ(Q) displays the typical increases observed in other glass-formers liquids^28, 29^. What is remarkable, however, is the fact that the flux influences the Q-dependence of τ only via a Q-*independent* multiplicative factor, see Fig. 4. This indicates that the resulting observed dynamics can still be used to probe the Q-dependence of the real dynamics.

## Discussions

The intriguing findings described above suggest the existence of a surprising system-dependent material-radiation interaction which can completely alter the outcome of an experiment and great care should be taken when analyzing X-ray data. In the case of in vitreous SiO_2_ and GeO_2_, the interaction with X-rays leads to a dynamic process whose main features can be summarized as follows:

i) The incident X-ray flux induces an atomic motion at temperatures well below T_g_. The time scale for this motion is inversely proportional to the photon flux. No decorrelation is observed if the system is not irradiated (at least on the scale of hours).
ii) At fixed flux, the dynamics remains *stationary* and is independent of the accumulated dose.
iii) The decay time depends in a *reversible* way and *almost instantaneously* on the incident flux.
iv) The shape of the correlation functions is independent of the flux and should therefore reflect an intrinsic property of the glass.
v) The flux does not affect the wave-vector dependence of the decay time at least for intermediate and small Qs.
vi) The described induced dynamics is observed for simple oxide glasses. Different glasses display distinct behaviours, in that, for instance, metallic glasses are *not affected* by the X-rays.

The above observations point to a complex beam-activated process which differs from the classical radiation damage reported in XPCS studies on soft materials^30, 31^. In these cases, the dynamics clearly varies with the global dose, resulting often in pronounced aging phenomena that are usually triggered by significant structural damage, although there are cases where clear structural changes seem to be absent. The latter situation seems to imply the existence of a threshold for radiation damage that is lower for dynamical studies than the one for structural investigations, in agreement with recent studies of protein diffusion^32^.

In our work, the probed glasses show only a weak, almost negligible, structural change within the *global* irradiation dose used for the measurements (less than ≈10^4^ s at F_0_), see Supplemental Information, while we do observe remarkable modifications for larger accumulated dose. For this large dose the X-rays modify the average local structure leading to a decrease of the intensity of the maximum in the static profile and a concomitant increase at ≈0.9 Å^−1^ (see Figs S4 and S5). Despite the occurrence of strong radiation damage at larger accumulated doses, we do not associate the observed dynamics to that damage: If it were the case, the dynamics would evolve with the accumulated dose (and thus the decay time as well) and would not be reversible when the flux is changed (see also Figs S6 and S7 for further confirmation).

Instead we believe that the effect reported in this work is likely due to *radiolysis*. Here the interaction with the X-rays generates localized electronic excitations with energies well below those necessary for knock-on events but large enough for atomic displacements^4, 33^. In order to convert these excitations into a mechanical response, they should have a lifetime of ≈1 ps and couple to the phonons. The details of these interactions are most probably system specific and thus would require additional computational modeling and experimental studies which go behind the purpose of this work. Metallic glasses do not show the discovered dependence on the flux since their electronic excitations delocalize faster, *i.e*. on the fs timescale. In alloys the atomic motion can be induced only through a direct transfer of momentum and energy by knock-on processes which require energies much higher than those employed here^12, 17^. In addition we point out that the glassy state of amorphous alloys cannot be considered *arrested* as in oxide glasses: It is indeed well known from diffraction studies that metallic glasses display structural atomic changes well below the glass transition temperature^21–23^. In this case XPCS directly probes the effect of these changes on the atomic motion^21^ which can lead to very *complex dynamical patterns under the same irradiated conditions* ^34, 35^. We do expect, however, that the flux induced dynamics found in the present work is likely to occur also in other non-conducting systems like polymeric compounds and molecular glasses, i.e. that this is a phenomenon that is not just a particularity of the simple oxide glasses considered here.

The induced dynamics in the simple oxide glasses strongly resembles the one observed in electron transmission microscopy^18^ and hints that the X-rays should not only be considered as a spectator but also as an important actor in the probed dynamics. Depending on the competition between the intrinsic and the induced dynamics, this effect can certainly become an issue for the determination of the associated time scales, as in the case presented here. However, one should keep in mind that it can be also considered as a novel type of pump and probe experiment that gives the *great opportunity to probe physical properties* of materials which cannot be achieved by means of any other technique.

Finally, it is worth highlighting that these beam-induced effects will become increasingly relevant for experiments at next generation synchrotrons^36, 37^ and at free-electron lasers sources^38^. In the latter case, techniques using high intense coherent beams, such as XPCS, are based on fs pulses that have intensities as high as current synchrotron sources provide in one second^39–44^. It is therefore likely that the above effects will be greatly amplified by non-linear responses of the system to collective excitations induced by the absorption of very short and intense X-ray pulses.

## Methods

### Sample preparation

Samples of the oxide glasses were prepared in the form of disks of 5 mm diameter by cutting bulk material with a diamond drill bit. The disks were mechanically polished to a thickness of 50 μm for SiO_2_ and of 20 μm for GeO_2_, in order to get the best compromise between the scattering signal and the speckle contrast. Bulk SiO_2_ was a commercial grade Spectrosil (SiO_2_-bis from Suprasil F300 with less than 1 ppm OH), while GeO_2_ was prepared by the usual melt-quenching procedure. Zr_65_Cu_27.5_Al_7.5_ metallic glasses were prepared by melt spinning in the University of Göttingen. The resulting ribbons had a thickness of about 40 μm.

### XPCS measurements

We performed several XPCS experiments at the beamline ID10 at ESRF by using 8.1 KeV radiation produced by three undulator sources. The coherent part of the beam (8 μm × 10 μm V × H full width half maximum) was selected by rollerblade slits placed upstream of the sample. The incoming intensity at the sample position was monitored continuously with a scintillation detector counting the photons scattered by air. The absolute incident flux is estimated by normalizing to scattered intensities from kapton foils^45^. The samples were inserted in a homemade resistively heated furnace mounted on a diffractometer in horizontal scattering geometry. The coherently scattered photons (speckles) were recorded by an Andor CCD device installed at ≈70 cm from the sample on a detector arm that was rotating around the sample to cover the Q–range 0.3–4 Å^−1^. Correlation functions were obtained following the analysis described in ref. 46. Oxides glasses were measured at room temperature while Cu_65_Zr_27.5_Al_7.5_ was annealed at T = 413 K (thus at T/T_g_ = 0.59). The choice of this temperature was dictated by the requirement to place the system in the temporal window where the decay time remains constant and sufficiently fast with τ ≤ 10^3^ s in standard working conditions at maximum flux (8.1 keV, no attenuators). This situation can be achieved by working at high temperature in the glassy state, where metallic glasses display stationary dynamics, likely related to an intermittent mechanism of aging^34^. At lower temperatures the dynamics is dominated by the typical fast aging of rapidly quenched metallic glasses^17, 21^ making the test extremely challenging as it would be difficult to disentangle X-rays induced effects and spontaneous changes of the decay time related to aging. For all samples, the measured contrast was ≈2–5% depending on the experimental conditions.

## Electronic supplementary material

Supplementary Information
