# Supplementary material for: Hard X-rays as pump and probe of atomic motion in oxide glasses
Source: Sci Rep. 2017 Jun 21;7:3962. doi: 10.1038/s41598-017-04271-x (PMC5479813; doi:10.1038/s41598-017-04271-x)
Supplement: Supplementary file 1 — Supplementary Information [file 41598_2017_4271_MOESM1_ESM.pdf]

SUPPLEMENTARY INFORMATION

Hard X-rays as *pump* and *probe* of the atomic motion in oxide glasses

Authors:
B. Ruta[1,2*], F. Zontone[1], Y. Chushkin[1], G. Baldi[3], G. Pintori[3], G. Monaco[3], B. Rufflé[4], and W. Kob[4]

[1)] ESRF- The European Synchrotron, F-38043, Grenoble, France.
[2)] Institut Lumière Matière, UMR5306 Université Lyon 1-CNRS, Université de Lyon, 69622 Villeurbanne Cedex, France.
[3)] Dipartimento di Fisica, Trento University, I-38123 Povo, Trento, Italy.
[4)] Université de Montpellier and CNRS, Laboratoire Charles Coulomb, UMR 5221, F-34095, Montpellier, France

1. X-ray induced atomic motion in GeO_2 at room temperature

Figure S1a shows the intensity auto-correlation function measured in vitreous germania for different values of the incident flux *F*. As it is the case for SiO_2, the decay time shifts toward faster time scales if the flux is increased. If the data is plotted as a function of time *t* times the mean flux, the different data sets superimpose (Fig. S1b).

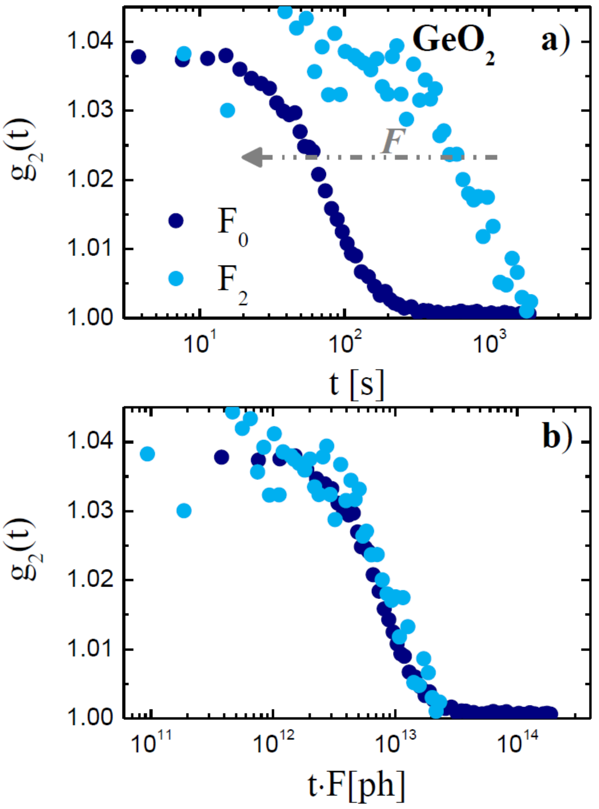

Figure S1: X-ray induced dynamics in vitreous GeO_2. (**a**) Intensity auto-correlation function measured in vitreous germania at T=295 K and Q_p=1.5Å^{-1}. Left curve (blue): $F_0 \approx 1\cdot10^{11}$ ph/s. Right curve (cyan): $F_2 \approx 1.2\cdot10^{10}$ ph/s. (**b**) Same data rescaled for the incoming flux.

This result implies that the decay time is completely fixed by the exposure time and that data taken with the same exposure time Δt_e but different sleeping time Δt_s perfectly overlap when rescaled for the mean flux impinging on the sample (Fig. S2).

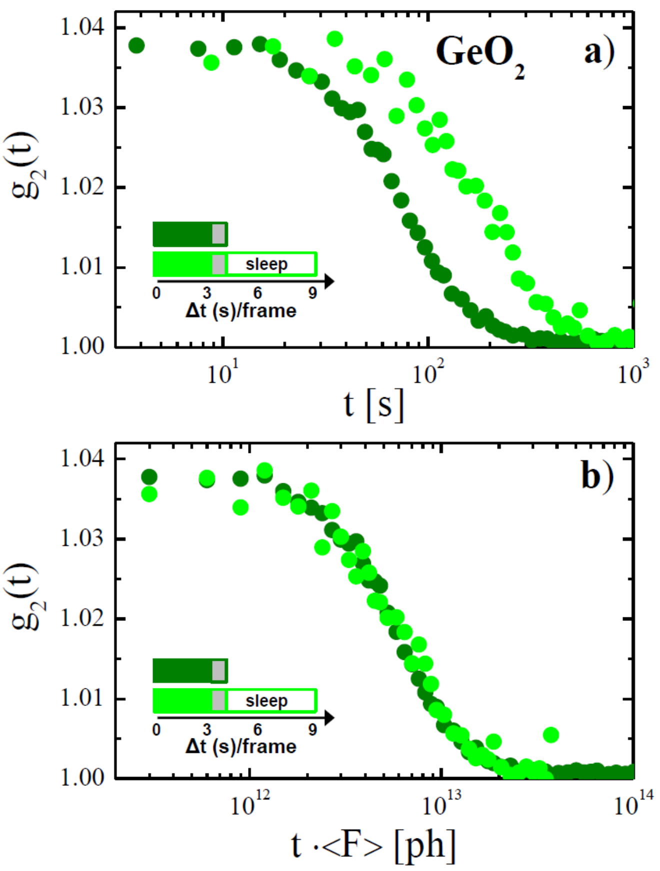

Figure S2: Characterization of the induced motion. (**a**) Intensity auto-correlation functions measured in vitreous germania at T=295 K and Q_p=1.5Å^{-1} with fixed exposure time per frame, Δt_e = 3 s, without (dark green) and with (light green) sleeping time Δt_s=5 s between frames. (**b**) Same data as a function of the time times the mean flux $<F>=F_0 \cdot \Delta t_e/\Delta t$.

Despite the presence of this flux-dependence of the relaxation time, the decay time does reflect the intrinsic properties of the system and varies with the probed wave vector Q, displaying an increase at low Qs as it is the case in SiO_2 (Fig. S3).

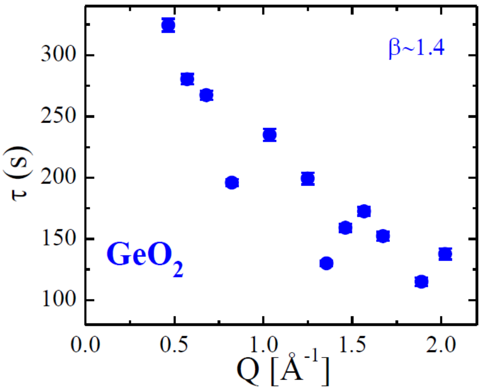

Figure S3: Wave-vector dependence of the atomic motion. Wave vector dependence of the characteristic decay time in vitreous germania measured at T= 295K and for $F_0 \approx 1 \cdot 10^{11}$ ph/s (blue circles).

2. Effect of the X-rays on the structure and reproducibility of the data

By irradiating always the same spot we do observe eventually a structural change in SiO_2. The resulting damage occurs slowly and it is basically negligible for short global irradiated times, i.e. the times considered in the present work, while it becomes significant after $\approx 10^4$s of irradiation with maximum flux F_0 at 8 keV. For larger irradiation times, there is a drop of the intensity at the first maximum of the static structure factor S(Q) which is accompanied by an increase at wave-vectors around Q=0.6 $Å^{-1}$. This is shown in Fig. S4 where we report the intensity static profile measured for different global accumulated dose.

The fact that the damage is very slow at the beginning explains while we do observe stationary and reversible dynamics during the measurements reported in the main manuscript.

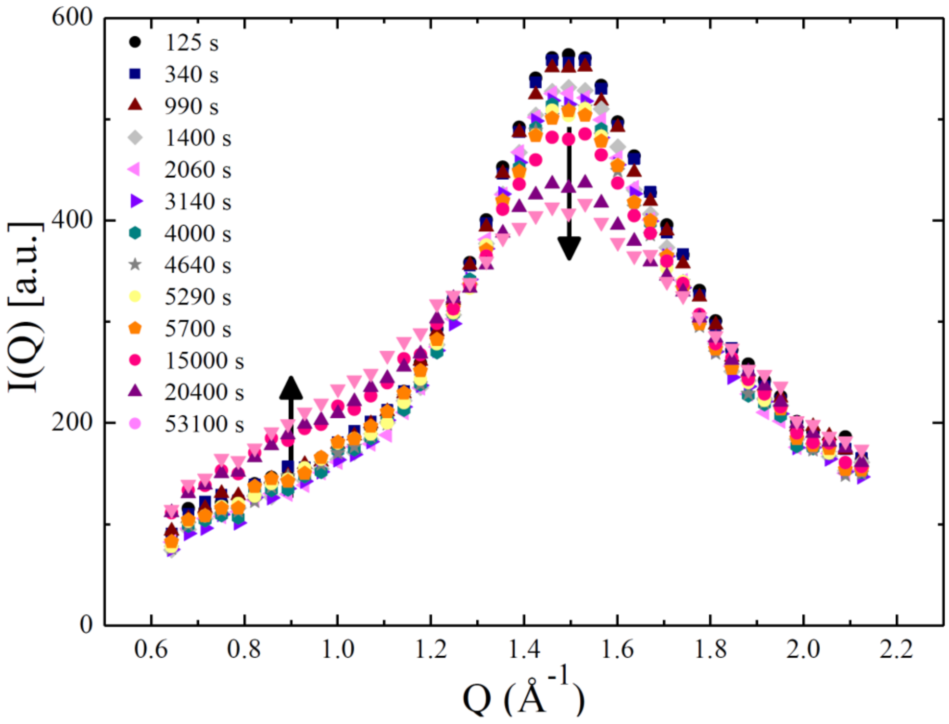

Figure S4: Effect of the X-rays on the structure. Static intensity profile measured in vitreous silica at T=295 K for different global irradiated times. The data are normalized by the incoming intensity after background subtraction. The arrows indicate the loss of intensity at the first maximum and the concomitant increase at around 0.9 $Å^{-1}$ due to the X-ray irradiation.

In order to characterize this radiation damage effect quantitatively we have fitted the intensity profile with a Lorentzian and in Fig. S5 we show the so determined fit parameters obtained from samples with different irradiation conditions. Purple circles are taken by irradiating always the same spot with F_0. Orange squares are measured always on the same spot with F_1, while magenta stars are measured with F_1 on different spots which were previously irradiated to measure the dynamics reported in Fig. 5 of the main manuscript (Q dependence measured with F_1). From this figure it is clear that the irreversible damage becomes significant only for global irradiation dose higher than 10^4 s at maximum flux F_0.

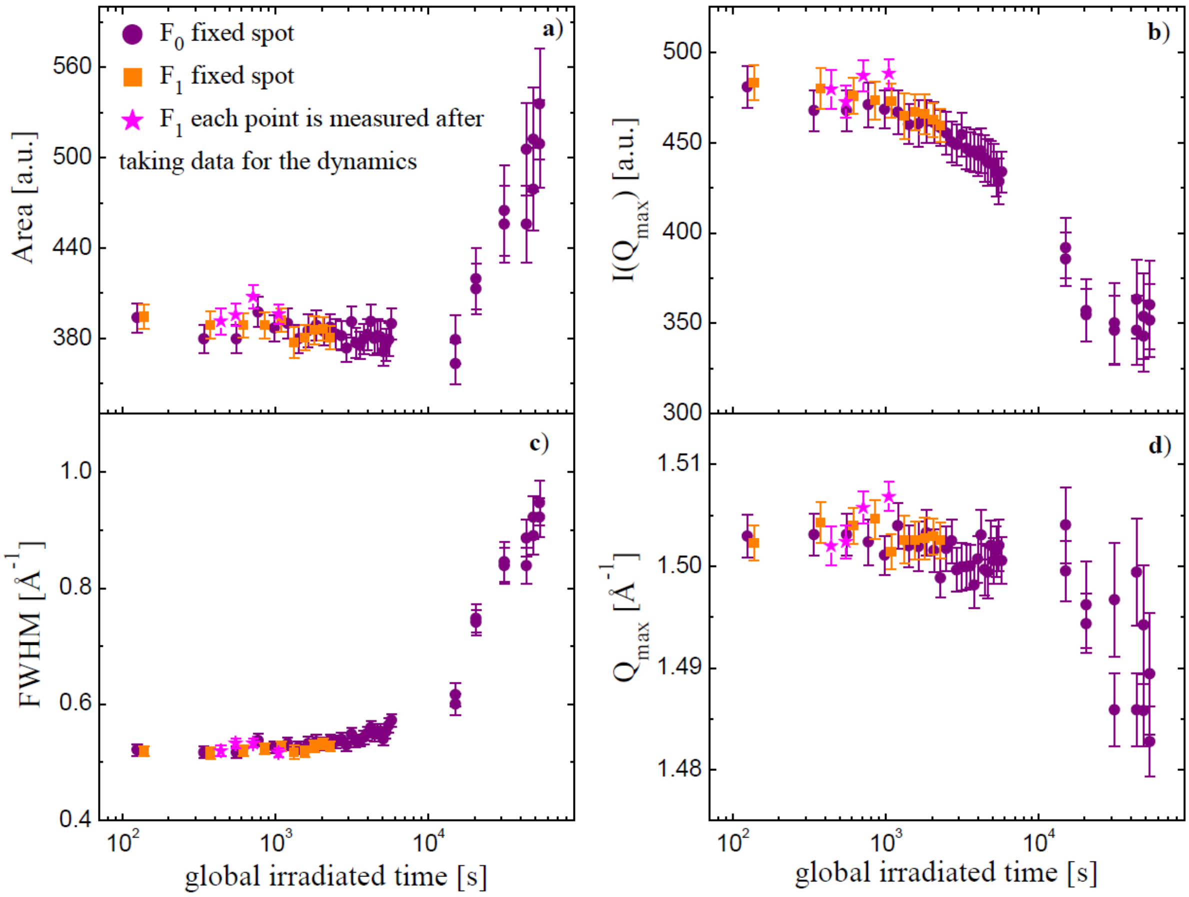

Figure S5: Characterization of the structural damage. Integrated area (**a**), intensity of the maximum (**b**), full width at half maximum (**c**), and position of the maximum (**d**) of the static intensity profiles measured in vitreous silica at T=295 K and as a function of the global irradiated times. Purple circles are data taken with F_0 on a fixed sample position. Orange squares are measured with F_1 always on the same sample position, while magenta starts have been collected with F_1 after taking the dynamical data reported in Fig. 5 in the manuscript.

The absence of a signature of the structural damage in our XPCS dynamical data is confirmed by the reversibility and the perfect scaling in the data reported in the main text and is also shown in Fig. S6 where we do report on the left the two-time correlation function measured during an acquisition with maximum flux. The broadening of the intensity along the main diagonal remains constant during the X-rays illumination demonstrating that no damages are occurring in the material. This is further confirmed by the fact that the number of photons collected by the detector follows exactly the behaviour of the incoming intensity (panel b).

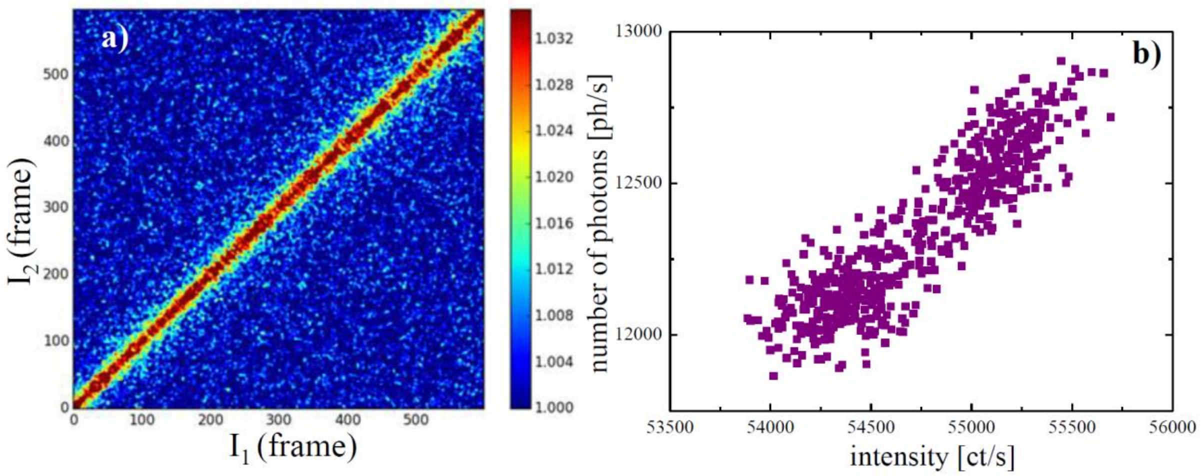

Figure S6: Absence of structural damage and stationary dynamics for low global accumulated dose. (**a**) Two-time intensity correlation function measured in SiO_2 at T=295 K and Q_p=1.5Å^{-1} with full intensity F_0. (**b**) Corresponding number of scattered photons impinging in the detector as a function of the incoming intensity per frame. The lack of beam damage is confirmed by the straight correlation between these two quantities.

Figure S7 shows the reproducibility of the data with global irradiated dose up to ≈10^4 s. This confirms the independence of the data reported in the manuscript with respect to the global dose and thus the absence of structural damage on this time scale.

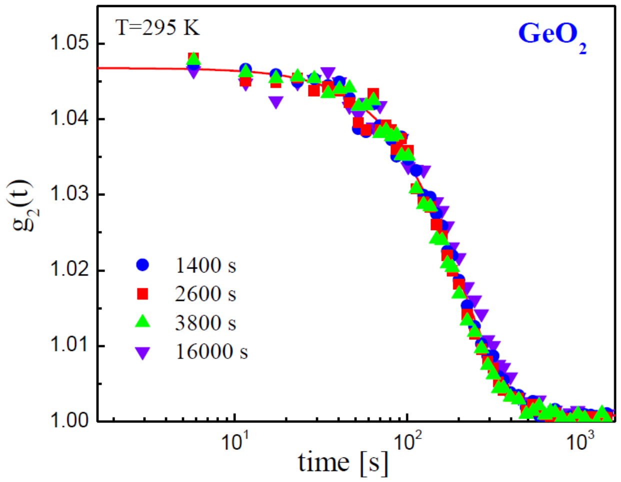

Figure S7: Reproducibility of the data for low global accumulated dose. Correlation functions measured in vitreous germania at T= 295K and for maximum flux $F_0 \approx 1 \cdot 10^{11}$ ph/s. The data are taken by irradiating always the same spot with increasing global dose. The dynamics remains stationary within the explored irradiation time. The line is the best fit with the KWW model function giving τ~300±20s at 8keV with no attenuators and a shape parameter β~1.5±0.1.
